# Audit of Risk Assessment Tool in Adolescent Eating Disorders

**DOI:** 10.1192/bjo.2024.620

**Published:** 2024-08-01

**Authors:** Mallika Punukollu, Camilla Mulligan

**Affiliations:** NHS, Glasgow, United Kingdom

## Abstract

**Aims:**

Aim:

To develop better prioritisation and assessment of high-risk patients.

Standard:

RCPsych Junior MARSIPAN guidelines advise that reasonable aims for a first presentation to primary care involve physical examination and referral to the appropriate CAMHS or paediatric service depending on the level of risk. The framework set out within the guidelines can be used to highlight areas useful to assess and grade concern.

NICE guidelines also state that in primary care, when assessing for an eating disorder or deciding whether to refer people for assessment, the following should be considered: BMI or body weight for their age, rapid weight loss, restrictive eating practices, mental health problems, physical signs of malnutrition, poor circulation and behaviours such as excessive exercise, unexplained electrolyte imbalance or hypoglycaemia.

Indicator and target: Proportion of GP referrals which contain information to allow risk assessment and prioritisation of referrals within CAMHS.

**Methods:**

Eating disorder referrals were reviewed over a three month period. Using EMIS, SCI store referrals were reviewed to check the source of referral and information included.

Referrals were checked against the following criteria: weight, degree of weight loss, fluid intake, calorie intake, heart rate, blood pressure, mental health screening, compensatory behaviours and blood analysis.

**Results:**

First cycle Results: from period from May–July 2021, comprising 8 referrals. The percentage of referrals which included information on the following were: weight 25%; weight loss 50%; calorie intake 37.5%; fluid intake 12.5%; heart rate 0%, blood pressure 12%, mental health 62%, compensatory behaviours 12.5%, bloods analysis 25%.

Action plan: Pro forma was developed to increase awareness of the information that is useful to CAMHS to prioritise and assess risk as per standards expected from NICE and Junior MARSIPAN guidelines.

This was circulated to East Renfrewshire GP practices in August 2021.

Second cycle Results: Referrals were reviewed in the period after the pro forma was circulated from Sept–Dec 2021. This comprised of 4 referrals. Upon review results showed that the percentage of referrals which included information on the following were: weight 100%; weight loss 50%; calorie intake 25%; fluid intake 25%; heart rate 25%, blood pressure 50%, mental health 100%, compensatory behaviours 75%, bloods analysis 50%.

**Conclusion:**

The audit showed, that following the pro forma being circulated, there was an improvement in proportion of referrals which contained appropriate information (26% compared with 55% cumulative percentage of information included).

